# Impact of Extracellular Matrix on Cellular Behavior: A Source of Molecular Targets in Disease

**DOI:** 10.1155/2015/482879

**Published:** 2015-11-05

**Authors:** Spyros S. Skandalis, Katalin Dobra, Martin Götte, Evgenia Karousou, Suniti Misra

**Affiliations:** ^1^Biochemistry, Biochemical Analysis & Matrix Pathobiology Research Group, Laboratory of Biochemistry, Department of Chemistry, University of Patras, 26500 Patras, Greece; ^2^Karolinska Institutet, Department of Laboratory Medicine, Division of Pathology, Karolinska University Hospital, F-46, 141 86 Stockholm, Sweden; ^3^Department of Gynecology and Obstetrics, Münster University Hospital, 48149 Münster, Germany; ^4^Department of Surgical and Morphological Sciences, University of Insubria, 21100 Varese, Italy; ^5^Department of Regenerative Medicine and Cell Biology, Medical University of South Carolina, Charleston, SC 29425, USA

In the last few decades, outstanding strides have been made on understanding the impact of extracellular matrices (ECMs) on cellular behavior in health and disease. It is now clear that ECMs are not inactive space-filling materials but, in contrast, apart from their structural roles they interact with cells and generate signals to control a multitude of vital cellular functions. Currently, there are strong indications that ECMs could potentially play a groundbreaking role in drug discovery since they comprise an invaluable source of multiple molecular targets. The established key roles of specific ECM effectors, such as proteoglycans, hyaluronan (HA), biosynthetic enzymes, cytokines, and growth factors, in the development and progression of several diseases suggest that ECMs hold a great potential in driving the design and development of novel disease targeting tools.

The focus of this special issue is to highlight the role and impact of specific ECM effectors on cellular behavior as well as their potential targeting that could advance the treatment of various diseases, such as skeletal and skin disorders, fibrosis, and cancer. It consists of two original research papers and four review articles covering a broad range of topics.

S. Albeiroti et al. in their paper entitled “Hyaluronan's Role in Fibrosis: A Pathogenic Factor or a Passive Player?” point out critical parameters that link HA to fibrosis and discuss the role of HA as well as its cellular receptors and HA anabolic/catabolic enzymes in different fibrotic diseases. The presented data suggest that HA and its regulatory pathways potentially represent novel targets for antifibrotic therapies.

A. Korpetinou et al. in their paper entitled “Increased Expression of Serglycin in Specific Carcinomas and Aggressive Cancer Cell Lines” evaluate the expression of the proteoglycan serglycin in several cancer cell lines and tissues and find that serglycin is expressed at high levels in more aggressive cancers. This experimental study suggests that the overexpression of serglycin by cancer and stromal cells may augment the expression of inflammatory mediators and proteases affecting the behavior of both stromal and cancer cells and providing a novel molecular target in aggressive cancers.

M. A. Soares et al. in their article entitled “Heparan Sulfate Proteoglycans May Promote or Inhibit Cancer Progression by Interacting with Integrins and Affecting Cell Migration” discuss the role of the interplay between integrins and heparan sulfate proteoglycans (such as syndecans and basement membrane proteoglycans) in health and cancer progression. This review highlights the need of further analysis and deeper understanding of the functions of integrin-heparan sulfate proteoglycans interactions in cancers in order to develop novel treatments based on analog molecules or prognostic factors that would be beneficial to patients.

The review by S. Mizumoto et al. entitled “Mutations in Biosynthetic Enzymes for the Protein Linker Region of Chondroitin/Dermatan/Heparan Sulfate Cause Skeletal and Skin Dysplasias” focuses on the recent advances in the study of cartilage and connective tissue disorders caused by defects in the biosynthesis of the common glycosaminoglycan-protein linker region tetrasaccharide in proteoglycans, called glycosaminoglycan linkeropathies. The authors describe the mutations of the glycosyltransferases responsible for the biosynthesis of the linker region and suggest that a deeper understanding of the molecular pathogeneses of glycosaminoglycan linkeropathies may lead to the design of new therapeutics for these diseases.

The review by B. Gidwani and A. Vyas entitled “A Comprehensive Review on Cyclodextrin-Based Carriers for Delivery of Chemotherapeutic Cytotoxic Anticancer Drugs” summarizes the advantages of the cyclodextrin-based nanotechnology for effective delivery of anticancer drugs aiming at minimizing off-target effects observed with other drug delivery systems.

H. Pratsinis and D. Kletsas in their paper entitled “Organotypic Cultures of Intervertebral Disc Cells: Responses to Growth Factors and Signaling Pathways Involved” use a 3D culture system in order to study intervertebral disc (IVD) degeneration. They investigate the response of IVD cells in 3D organotypic gels to growth factors as well as the signaling pathways involved and propose that these culture systems may be useful for the design of novel regenerative therapies of degenerated IVD.

In conclusion, several emerging issues related to the critical roles of ECMs molecules in the regulation of cell behavior are presented in this special issue. We hope that this issue will add to the rapidly expanding field of matrix pathobiology underlying specific diseases and will help in the development of matrix-based therapeutic strategies in the near future.

